# Self-sealing posterior scleral perforation in airgun ocular trauma, surgical tip: a case report

**DOI:** 10.1186/s12886-020-01435-8

**Published:** 2020-04-22

**Authors:** Fabiana Mallone, Michela Marcelli, Riccardo Monsellato, Federica Franzone, Magda Gharbiya, Alessandro Lambiase

**Affiliations:** Department of Organ of Sense, Sapienza University of Rome, Viale del Policlinico, 155 00161 Rome, Italy

**Keywords:** Ocular trauma, Vitreous hemorrhage, Airgun, Pellet, Case report

## Abstract

**Background:**

Intraorbital metallic foreign bodies have varied clinical presentations. Here, we report the unusual case of intraoperative evidence of spontaneously healed posterior scleral perforation in a severe ballistic trauma without previous instrumental signs of penetrating wound and complete visual restoration after surgery.

**Case presentation:**

The patient was hit by several lead hunting pellets in the chest, abdomen, limbs, face and orbit. Computed Tomography (CT) images revealed the presence of a pellet within the orbitary cavity, close to the optic nerve, with no signs of penetrating ocular wound. While performing vitrectomy for severe vitreous hemorrhage, a point of strong adherence between a old hemorrhage and retinal surface was identified and managed conservatively, as it was attributed to trauma related-impact area. So, lead foreign body took an unusual trajectory impacting the globe and finally lodging back in the deep orbitary cavity, in absence of significant ocular injury and with visual prognosis preservation.

**Conclusions:**

Our findings provide further information on orbital injuries from airguns, a theme of growing popularity and concern. Intraoperative recognition of hardly removable old hemorrhagic clot as self-blockage site of posterior scleral penetrating trauma, allowed for surgical stabilization and minimal solicitation of the area to avoid inadvertent perforation.

## Background

In the recent past, like-gun shot wounds have become a growing clinical concern due to the increasing popularity of airguns, with advances in compressed-gas technology leading to a significant increase in power and velocity of these weapons [1, 2].

Common post-traumatic orbital injuries include anterior chamber injuries, injuries to the lens, open-globe injuries, ocular detachments, intraorbital foreign bodies, carotid cavernous fistula, and optic nerve injuries [3, 4]. More in detail, retinal oedema and haemorrhage occur with an estimated frequency of 49.5% among major injuries, hyphaemia 59%, and vitreous haemorrhage 41% [1]. The use of imaging techniques, along with comprehensive ophthalmologic examination, is crucial in assessing traumatic globe injuries. Computed Tomography (CT) scanning proves as the most sensitive and readily available imaging approach, and notably allows for detection of optic nerve damage, foreign bodies and fractures, with related prognostic information in terms of visual outcome. Ultrasonography (US) is fast and safe procedure, and shows potential benefit for evaluating the orbit, the globe and associated contents; however US is contraindicated if there is suspicion of a ruptured globe. Magnetic resonance imaging is not characteristically performed in an emergency setting; and it is contraindicated if the suspected foreign body is ferromagnetic [5]. Rapid assessment and examination following trauma are important as timely intervention may save vision.

## Case presentation

**Fig. 1:**
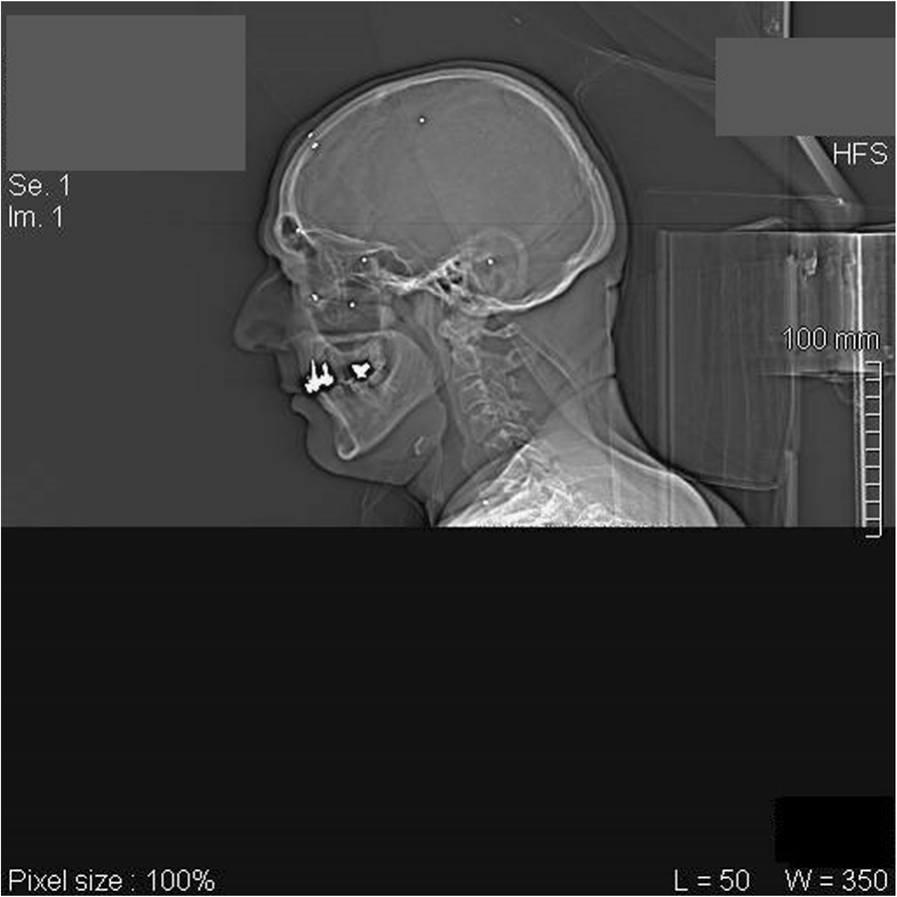
CT scan Sagittal Recontruction: head localization of numerous lead pellets

A 58-year-old man, victim of airgun aggression, presented to our General Emergency Unit of the Hospital Umberto I of Rome, ‘Sapienza’ University with multiple wounds such as entry holes in the subcutaneous space and in muscles of thorax, abdomen, limbs, groin and face (Fig. 1).

**Fig. 2:**
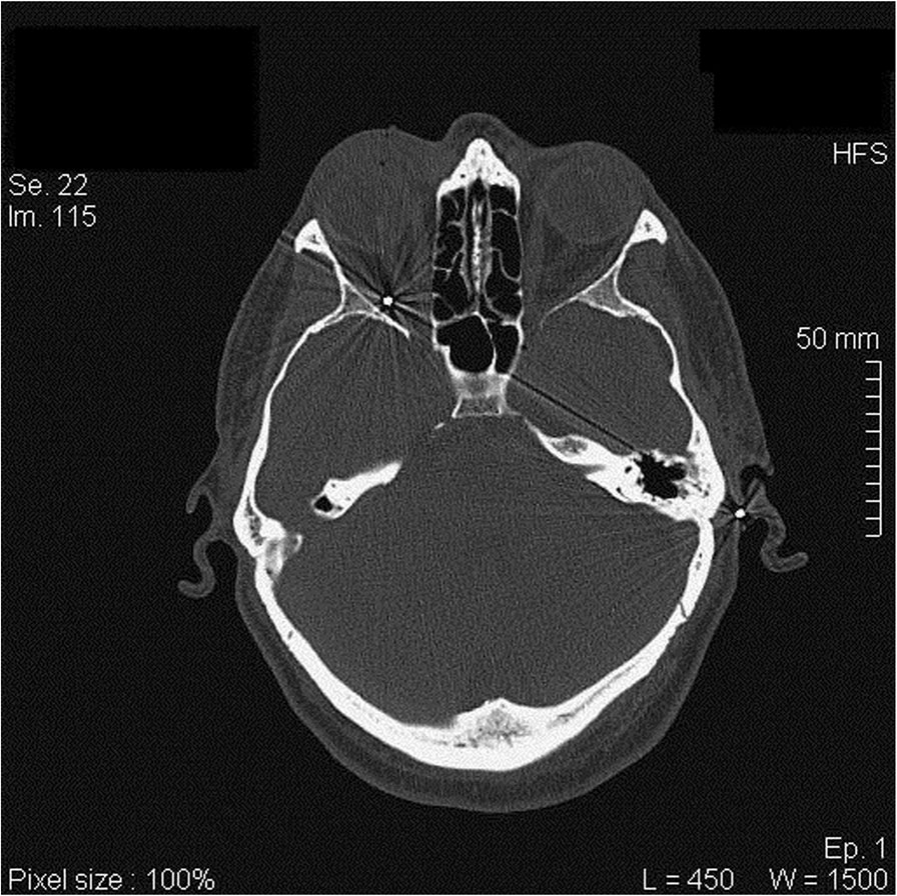
CT scan Axial Reconstruction: identification and localization of the intraorbital lead pellet


He underwent total body CT and no vital organs resulted damaged. Blood tests were within normal limits and the patient was fully conscious, well oriented, without signs of urgent surgical need. As far as his orbital involvement, it was described full thickness wound in his right upper eyelid, swelling and ecchymosis in his right orbital region, accompanied with important vision decline consequently to massive vitreous hemorrhage in the right eye. CT scans of the orbital region revealed the presence of a hunting lead pellet within the orbitary cavity, supero-temporally close to the optic nerve, in the deep retrobulbar space between the optic nerve and the lateral rectus muscle, with no penetrating wound of the globe (Fig. 2). The patient was evaluated by complete ophthalmological examination including measurement of the best-corrected visual acuity (BCVA), intraocular pressure, slit lamp biomicroscopy, mydriatic indirect fundus biomicroscopy under tropicamide 1%, ocular ultrasound and ocular ultrabiomicroscopy (UMB). The patient could not perceive light with the injured eye, ocular motility was normal, the anterior chamber was formed, and optically empty, intraocular pressure was within normal limits (14 mmHg) and a right afferent pupillary defect was observed. Additionally, inferior subluxation of lens and traumatic cataract were noted, and fundus details were not visible because of media opacities. UBM exam confirmed the inferior crystalline subluxation demonstrating a relative integrity of the ciliary bodies-zonula complex. Moreover, blood thickening and clots in vitreous cavity with tractive attitude on the retina in the central superonasal sector, were described. We ultrasonographically observed no retinal detachment, no penetrating wound, no deviation of the optic nerve, no orbital identification of the pellet because too deep in the muscular cone. While performing vitrectomy for dense hemovitreous 3 weeks after injury, we created 360-degree circumferential hyaloidectomy at the level of the midperiphery, then we slowly trimmed the posterior vitreous inducing a hyaloid detachment to separate it from retinal surface. During surgical procedure, the vitreoretinal surgeon noticed that a fibrin mass was tenaciously attached to the retinal plane, along the superonasal arcade. In particular, it resulted not excisable by using the vitrectome, unlike the whole blood vitreal content that was totally removed. Fibrin was therefore gently shaved as much as possible, and left in situ to avoid any risk of perforation. Argon laser treatment was then applied to delimit and stabilize the shot area. Phacoemulsification of cataract was performed too, and primary implantation of 3 pieces intraocular lens (IOL) into the ‘sac’ was possible because of adequate capsular and zonular support. (Video). The patient regained total visual restoration immediately after surgery. 20/20 BCVA was recorded at the last follow-up visit 1 month post-operatively, with residual inferotemporal visual field defect correspondingly to the area of bulbar impact.

**Additional file 1: Video.** 25 G Pars-plana Vitrectomy and IOL implantation. Intraoperative identification of the ocular site of impact and related management.


## Discussion and conclusions

The orbitary cavity is vulnerable to injury from airgun pellets and direct hits invariably result in severe structural and functional damage to the eye or other orbital contents. Foreign bodies can reach the orbital space because of bulbar double perforation or via lid lacerations through peribulbar tissues. In the latter way, as the globe integrity is maintained, visual acuity is preserved [6–8].

Here we report the intraoperative identification of a fibrinous mass, acting as plug, resulting hard to be removed by the use of vitrectome, and recognized as the ocular point of impact of an intraorbital lead pellet. On the basis of clinical features and radiological appearance of the foreign body, we speculate that it traversed the orbital septum through the right upper lid and peribulbar soft tissues, and superonasally impacted the eye without being able to penetrate it. The airgun pellet then definitely localized itself at the bottom of the orbitary cavity, superotemporally to the orbital apex and medially to the lateral rectus muscle. This lucky trajectory preserved the eye, the optic nerve and the whole orbitary content from severe and irreversible structural and functional damage. The massive vitreous hemorrhage and lens subluxation were surgically managed, and the fibrin mass was maximally reduced, but not further solicited, in order to prevent any risk of unintentional perforation. We decided not to remove the intraorbital pellet as it was not readily accessible, and the patient did not complain any orbital complications such as orbital cellulitis or systemic toxicity. About that, most metals retained in the orbit, including lead, are inert and in the absence of infection cause no disturbance and should be managed conservatively in the absence of specific indications for removal [9, 10].

We immediately judged this case as unique for the clinical scenario and surgical management. A few articles in Literature are based on this topic, especially when the pellet is located posterior in the orbit and injury occurs without permanent visual impairment [11–13].

We believe in the importance of disclosing the information contained in this report and relative multimedial material, in order to promote exact recognition of ocular trauma point in case of a fibrin plug resulting hardly removable using vitrectome, and so to avoid any potential induced perforation. The combination of documented intraoperative evidence of ocular site of impact, absence of any instrumental data of penetrating wound and total visual recovery, represented an exceptional occurrence and no similar cases have been published in Literature hitherto.

## Data Availability

The data used to support the findings of this study are available from the corresponding author upon request.

## Abbreviations

CT: Computed Tomography
US: Ultrasonography
UMB: Ultrabiomicroscopy
BCVA: Best-corrected visual acuity
IOL: Intraocular lens
